# Graduate Biomedical Science Education Needs a New Philosophy

**DOI:** 10.1128/mBio.01539-17

**Published:** 2017-12-19

**Authors:** Gundula Bosch, Arturo Casadevall

**Affiliations:** Department of Molecular Microbiology and Immunology, Johns Hopkins Bloomberg School of Public Health, Baltimore, Maryland, USA; Albert Einstein College of Medicine

**Keywords:** Ph.D., education, graduate

## Abstract

There is a growing realization that graduate education in the biomedical sciences is successful at teaching students how to conduct research but falls short in preparing them for a diverse job market, communicating with the public, and remaining versatile scientists throughout their careers. Major problems with graduate level education today include overspecialization in a narrow area of science without a proper grounding in essential critical thinking skills. Shortcomings in education may also contribute to some of the problems of the biomedical sciences, such as poor reproducibility, shoddy literature, and the rise in retracted publications. The challenge is to modify graduate programs such that they continue to generate individuals capable of conducting deep research while at the same time producing more broadly trained scientists without lengthening the time to a degree. Here we describe our first experiences at Johns Hopkins and propose a manifesto for reforming graduate science education.

Imagination is more important than knowledge.

—Albert Einstein

## THE CURRENT SYSTEM IS IN NEED OF REFORM

In recent years, there have been numerous calls to reform graduate biomedical science education, particularly at the doctoral level (1–17). Currently, many institutions favor densely packed curricula with fast-paced instruction focused on detailed subject matter and a high test frequency in an attempt to shorten the time to a degree. Despite the risk of cognitive overload and low information retention rates, little room is left for skill training in critical thinking, creative problem solving, and putting what was learned into a larger context that creates meaning. Many current biomedical and health science programs are effective at educating individuals capable of carrying out deep scientific investigations in highly specialized areas; however, they often lack the breadth of expertise and skills necessary to allow their graduates to move to other fields of science or participate in the science-relevant areas needed by society at large. In essence, our biomedical education system tends to produce graduates best suited for postdoctoral training, which tends to further specialize them (3, 9, 18). Although numbers might be slowly declining (19, 20), many students in science, engineering, and health still report commitment to pursuing an academic research career (21). Yet only a subset are likely to be employed in academic occupations in the long term (3–5, 19–23), with the numbers of positions for tenure track faculty being on a decline over more than 2 decades (22, 24). In response to those trends, policymakers and some members of the research community have argued for a reduction in the number of students entering graduate schools (2, 3). However, we agree with those opinions in the field that emphasize the need for a stable scientific workforce because of a high demand for engaged science practitioners in many areas of society (19, 20, 23, 25–29). Besides academia, there are job options in such fields as industry, advocacy, government, education, communication, social work, and community service, etc. (10, 11, 14–17, 19–29). This complex ecosystem requires individual adaptability and “outside-the-box” thinking, as well as better career counseling and research training options on the pre- and postdoctoral levels (1, 3–12, 15–31).

Currently, scientific training is largely dependent on a local mentorship culture, which takes place for the most part in the form of apprenticeships during practical thesis work. The burden in this system falls largely on the individual thesis adviser to teach the skills of good research practice (1, 12). Unfortunately, given the variability in thesis projects and institutional environments and variability in the ability of mentors to teach the relevant material, the training experiences of individual students can differ significantly (32). Hence, there is no guarantee that this guild-like education system delivers the required instructional quality to all trainees (1).

Recently, several institutions and agencies, among others, the American Academy of Microbiology and the National Academies of Sciences, Engineering and Medicine (10, 11, 14–17), have reevaluated current problems of the biomedical sciences and made recommendations on how to better prepare graduate students, as well as their mentors, for the complex workforce requirements inside and outside academia. Suggestions for essential core competencies complemented other calls and initiatives for reform (1, 3–17, 28, 33–40). These included the need to enrich current graduate education with preprofessional competency training in skills required by academic and nonacademic sectors. Based thereupon, in this and earlier essays, we and others argue that graduate students in the biomedical disciplines need to be trained formally (as opposed to solely through individual mentoring by their thesis advisors) in interdisciplinary, critically philosophical thinking skills. We claim that the goal of true science education reform efforts should be to train and mentor a new leadership of practitioners, whose members are

- broadly interested, creative, and self-directed (9), as were some scientists in the era of Louis Pasteur, Marie Curie, Albert Einstein, and Linus Pauling (41–43);
- versed in epistemology, sound research conduct and error analysis, according to the “3R” norms of good scientific practice—rigor, responsibility, and reproducibility (12);
- skilled in reasoning using mathematical, statistical, and programming methods and able to tackle logical fallacies (10–12, 14–16);
- committed to high ethical standards, mentorship, and teamwork (7, 11–16);
- effective leaders, teachers, and communicators on the expert level, as well as with the public (33, 35);
- able to think innovatively and across disciplinary boundaries (18, 43–45); and
- aware of the diversity of societal tasks that need to be mastered by science practitioners today (19, 23).

Training modules on rigor and reproducibility, as well as other educational resources on scientific survival skills have been produced and are publicly available (46–49). However, the mere provision of materials is only a first step and, by itself, unlikely to be effective. Rather, they need to be implemented into specifically designed learning experiences, and their effectiveness needs to be evaluated by outcome-oriented educational research. Without validated guidelines on how to utilize available resources in the classroom, the quality of teaching practices can vary widely. This might arguably contribute to the widespread reproducibility issues that exist (32). Following recent funding initiatives by the National Institutes of Health (50–52), the National Science Foundation (53, 54), and the Burroughs Wellcome Fund (55), several educational institutions have engaged in efforts to modify and supplement existing graduate science programs (26, 36, 50). First evaluation reports from these efforts are being published (56).

## A “NEW” PHILOSOPHY BASED ON CLASSIC TENETS

Can there be “one” comprehensive educational concept to solve all of the issues mentioned above? Probably not. Graduate school orientations, missions, values, and organizational structures are too diverse to be homogenized into one standardized instruction model, similar to the “core curricula” or “distributional requirements” of many undergraduate institutions. In fact, such standardization may not be desirable on the graduate level since heterogeneity in educational approaches can lead to experimentation and innovation. One might even argue that one of the current problems in graduate education is a lack of diversity from program to program.

More than two centuries after the scientific revolution, we have learned much about what constitutes good science, but these insights are frequently not formally integrated into the regular curricula used to train scientists. Today’s doctoral education in the biomedical sciences is largely homogenized around a set of required courses, rotations in potential training laboratories, and laboratory work with relatively little room for educational experimentation. In going forward, one immediate step could be to define and test frameworks in some institutional settings that can inform and provide incentives for interested educators in other organizations. A repeating theme brought up among undergraduate and graduate science educators across all disciplines is the need for more critically reflective practice (57, 58). Since a critically rationalist approach, a term coined by Karl Popper (59), is at the heart of doing science, we agree with the suggestion that science education should be geared toward a stronger emphasis on philosophical elements, thereby putting the “Ph. back into Ph.D.” education (1, 9, 12, 60–63). Teaching graduate students how to critically “think and do science” could help to bridge the gap between the fundamentals taught in undergraduate institutions and the requirements of competency-based thesis work on the graduate level.

However, in our view, it is not sufficient to merely prescribe critically philosophic elements for inclusion in graduate curricula to reform the educational framework in science. Instead, what is needed is a combination of rationalism with critically reflective practice (64, 65); genuine meaning making in a relevant, active learning context (66–69); and passionate engagement (70) to create powerful learning experiences. To develop a mindset of reflective practice, students should have frequent opportunities to exercise the various facets of critical thinking, comprising challenging assumptions, evaluation and reasoning, creative problem solving, and communication (71, 72). To apply these skills in relevant contexts, previous reform efforts in higher education have emphasized how important it is to “teach science like we do science” (73, 74; see also http://www.biophysics.org/Publications/Newsletter/PastIssues/December2011/56thAnnualMeeting/tabid/3731/Default.aspx); besides becoming accustomed to rigorous research methodology, students need to develop an unvarnished, realistic view of the frequently erroneous decision-making processes and daily research practices in the biomedical sciences (73, 74). It is thereby critical to create an awareness of the epistemic limits of science (75, 76) and address error sources such as logical fallacies or sloppiness in experimental design (12, 13, 77, 78), just as much as cases of misconduct (79). On the other hand, there must be sufficient room for learning from the fun side of scientific blunders, serendipitous discoveries (45, 80), and success stories. To facilitate authentic experiences (69), learners could use self-chosen projects of their interest or aspects of their thesis work to apply central scientific concepts such as deductive and inductive reasoning. Moreover, students may be given opportunities to practice mindfulness, self-awareness, and communication skills in real-world situations such as community outreach settings (81, 82).

It is the sense of meaningfulness emerging from intrinsically motivated, active learning processes that provokes deep thoughts, sparks curiosity, and elicits the desire for self-directed exploration (83); and it is a learner’s passion that may lead to truthful and sustained commitment, potentially transformational experiences, and fulfilling lifelong careers (70, 84, 85).

## A PILOT PROGRAM AT THE JOHNS HOPKINS BLOOMBERG SCHOOL OF PUBLIC HEALTH

In the spirit of this philosophy of graduate science education, we describe here our early experiences implementing a novel Ph.D. track at the Johns Hopkins Bloomberg School of Public Health to help students experience how to broadly and critically “think science.” On the basis of an earlier essay (9), we called this approach the “R^3^ program,” referring to the norms of good scientific practice, the three R’s: rigor, responsibility, and reproducibility, that form the core of our educational efforts (http://www.jhsph.edu/departments/w-harry-feinstone-department-of-molecular-microbiology-and-immunology/academics-and-degree-programs/R3-PhD-program/index.html). A central goal is to preserve what works well in the current system, namely, in-depth laboratory training through hands-on mentored laboratory rotations and thesis work while reducing the density of highly specialized courses in the curriculum. Thus, we markedly decreased students’ overall course loads and partially filled the freed-up curricular room with protected time for critically creative reflection, collaborative problem solving, and dedicated time for practicums in settings outside academia (Tables 1 and 2). First-year courses emphasize practical aspects of epistemology, logic, reasoning, error analysis; and rigor and redundancy in experimental design; as well as ethics, integrity, and social responsibility. Fundamentals in probability, statistics, programming, and computational data analysis are taught with regard to the actual needs in students’ prospective lab environments (Table 1).

**TABLE 1:** R^3^ curricular plan for academic year 1: skill training in critically thinking and conducting science

Term 1^a^	Terms 2 and 3	Term 4
*How do we know? Theory and Practice of Science*^b^	*Critical Dissection of the Scientific Literature*	*Electives - Options*
Introduction to critical thinking in science (epistemology, logic, the 3R’s)	Primary literature journal club Interdisciplinary discussion series including case studies and “error detective games”	Rotation-oriented, subject matter courses to support thesis preparation Extradepartmental electives
*Anatomy of Scientific Error*^c^	*Practical Ethics in Science & Society I & II*	*Part-time Practicums - Options* Community outreach Science policy & advocacy Entrepreneurship Ethics in practice Science writing Teaching & mentoring Team skills Communicating with the media and the public
Errors versus misconduct in scientific practice	Questions of integrity and morality at theinterfaces of ethics and epistemology	
*Research Design* Practice-oriented, experimental methods and redundancy in research design Overview of laboratory research *Scientific Reasoning I* Introductory logic in data science; induction & deduction; correlation & causality Laboratory-rotation oriented vignettes and problem sets	*Training in Science Communication* Presenting effectively Writing for publication and funding Leadership through persuasive speaking Non-verbal communication *Scientific Reasoning II & III* Mathematical and statistical foundations and applications; programming basics Research-oriented, real-world cases and problem sets	

a At the Johns Hopkins Bloomberg School of Public Health, a term is 8 weeks long. All four terms include laboratory rotations.

b How do we know? Theory & Practice of Science; course information, https://courseplus.jhu.edu/core/index.cfm/go/about.schedule/coid/9185/.

c Anatomy of Scientific Error; course information, https://courseplus.jhu.edu/core/index.cfm/go/about.schedule/coid/9489/.

Many students (as well as practitioners) might feel unsure about how topics that do not appear to be immediately relevant to concrete subject matter, and procedural training could help them advance in their graduate careers or thesis research. The traditional overemphasis on teaching details and technical procedures at the expense of more generalized scientific competence may have the unintended consequence of creating the notion that memorized facts and technical skills are needed to successfully compete in the job market. However, given today’s ubiquitous internet access through computers and smart mobile devices, most individuals have almost immediate access to much of humanity’s accumulated knowledge. In a situation where knowledge is easily available, it may be more important to learn how to process this information than to commit details to memory. Consequently, the goal of the R^3^ program is to teach critically creative thinking, logical reasoning, and strategic decision making that would enhance students’ ability to valuably utilize the information that is readily available and assess its reliability. The current focus on overspecialization may contribute to insufficient communication skills, suffocation of exploratory curiosity and innovative potential, and the prevalent lack of appreciation of the actual “Ph.” in Ph.D. training (1, 9).

To build trust among students, educators, and mentors, a new curriculum like the R^3^ program needs to handle a balancing act; it needs to provide sufficient subject matter education such that students and their thesis advisers feel confident that the preparation received is sufficiently deep to enable students to successfully complete a thesis without lengthening training. At the same time, the curriculum must provide enough protected time to allow students to broadly and creatively explore their interests, engage in interdisciplinary dialogue, and explore alternative pathways outside academic research (5, 31, 36). To that end, we designed the first year of our R^3^ track (Table 1) by focusing on practically relevant, competency-building courses, while concurrent journal clubs and laboratory rotation schedules provide opportunities for continuous application. This curricular format allows students to explore and deepen their interests while providing real-world research examples. In addition, students make informed decisions about their fields of thesis research and connect with their future advisers and mentors early in the curriculum. Toward the end of the first academic year, the program provides room for a set of preselected subject matter electives. In agreement with the future thesis adviser, these electives convey introductory background knowledge and facilitate the transition into a student’s chosen field of thesis research. Moreover, protected time is built into the last weeks of the first academic year and the following summer break for students to explore fields of professional science practice outside academia, such as practicums in teaching, mentoring and leadership; science communication; business and entrepreneurship; science policy and advocacy; community outreach, or applied ethics.

Second-year training (Table 2) is less structured and puts a greater emphasis on professional competency training and enabling students to apply the skills learned in the first academic year to their thesis work. A wide array of discipline-specific learning options, e.g., in the form of self-study modules or preapproved open online courses, provide individualized, targeted opportunities to strengthen students’ subject matter basis in their chosen fields of thesis research. In communities of continuous practice, students intensify teaching, mentoring, and leadership competencies. Cross-disciplinary seminar series provide opportunities to communicate students’ work in preprofessional settings or at venues open to the public. Interdepartmental think tanks allow students to develop and debate innovative ideas for collaborations outside the narrow box of their thesis research field. In agreement with the thesis adviser, advanced graduate students may have additional opportunities for part-time career exploration inside or outside academic settings. Close collaboration between program directorship and participating faculty and the support of individual thesis advisers are of particular importance here (31).

**TABLE 2:** R^3^ curricular plan for academic year 2: continuous training in thesis-related background and professional practice skills

Training	Content, purpose	Terms^a^ offered
Thesis-oriented subject matter electives	Individual selection of learning options (regular coursework, preapproved, open online offerings, technical practice training, self-study) to strengthen students’ subject matter basis in their chosen field of thesis research	1–4
Innovation series	Seminars and distinguished lectures on “thinking science outside the box”	1–4, monthly
Communication	Continuous practice in communication skills for conversations with the public, media, and professional audiences; publications and grants; presentations; advocacy	1–4, biweekly
Team and leadership skills	Experiential competency development in emotional intelligence, cultural sensitivity and mindfulness, group dynamics, team citizenship and leadership, goal setting, conflict resolution, peer leadership	Periodical, 1–3-day workshops, flexible terms
Teaching and mentoring	Practice experience in teaching and mentoring, teaching assistance and instructional competency education, structured mentoring of younger students, assistance with thesis and career development	Flexible terms
Science history	Story-telling seminar series for faculty, fellows, students, and staff; narrative skills, community formation	1–4, monthly
Part-time practicums	Additional practicum and workshop experiences, offering opportunities to collect preprofessional insights into areas such as industry, education, and community outreach, administration, policy and advocacy, communication, writing, and publishing, etc.	Flexible terms
Mentored career planning and professional development plan	Peer-, alumnus-, and practitioner-mentored discussion communities; regular expert panels on career advising; compilation of individual development plan synchronized with thesis committee checkpoints	Continuous, 3 checkpoints/yr

a At the Johns Hopkins Bloomberg School of Public Health, a term is 8 weeks long.

At this early implementation stage, any experiences are naturally anecdotal and subject to change upon revision and improvement of the program. During our first pilot administration, enrollment comprised between 20 and 30 students per course, with participants from a variety of biomedical and health science disciplines. In every course, skill mastery is assessed through several lenses, such as discussion contributions, reflections, problem solving, project presentations, and peer feedback. To document individual student progression through the program and achievement of R^3^ core competencies, we selected a set of key course assessments, among others,

- a project describing revolutionary discoveries or important innovations in science, thereby evaluating their broader significance and historical or contemporary context;
- case study problems derived from real-world data that allow students to search for scientific errors and practice reasoning;
- thesis-related experimental design projects that imply demonstration of rigorous research methodology and justification of the design chosen;
- an argumentative debate of ethical dilemmas, including peer evaluation of the points made; and
- a project that requires oral and written communication of students’ thesis aspects to peers from other disciplines and the general public.

Every student will assemble a reflection-evidence-type portfolio (86) of their key assignments, composed over the duration of the program. Together with formal examinations due to accreditation requirements, a list of scholarly accomplishments, as well as evaluative observations by the thesis adviser, this system will provide a holistic picture of individual R^3^ students’ progression over time.

## A MANIFESTO FOR REFORMING GRADUATE BIOMEDICAL SCIENCE EDUCATION

Critical thinking in science needs to be accessibly conveyed to be thought provoking and motivating (75, 87, 88). Particularly advanced learners on the graduate and postgraduate levels need a clear practice orientation (83), which is not a trivial goal to achieve with an interdisciplinary audience. To meet as many of our learners’ needs as possible, we propose the implementation of a set of guidelines to help reform graduate biomedical science education. We acknowledge up front that the proposed manifesto is a compilation of previously established principles and theoretical frameworks for good educational practice. Although well known and adopted in other disciplines, they have not been widely implemented in graduate biomedical scientific training thus far, underlining the need for faculty support and mentoring (31).

### (i) Active learning strategies.

The flipped classroom model involves the use of short, recorded, conceptual presentations that students can follow at their own pace and time (89). This format reserves the majority of the contact time with instructors and peers for a range of active learning strategies, such as critical reflection, discussions, problem solving, and collaborative group work (66, 69, 87, 88, 90–94). Diversification of instructional and assessment methods allows accommodation of a wide range of learner needs and preferences (95) and may positively influence learning processes (96).

### (ii) Facilitating emotional and cognitive connections.

Being able to relate to the material taught is important for students (83). Supplementing the often technical primary and textbook literature on scientific topics through broadly applicable and accessibly written background resources can help open doors to subjects that otherwise might be difficult to connect to. For instance, editorials or op-eds, news and views columns of general science journals, podcasts, or TED Talk videos can help students relate theoretical science concepts to their life reality or current events (97). Moreover, the combination of role modeling (98) and storytelling (99–101) can be a powerful tool, e.g., by sharing personal experiences from one’s own scientific practice when discussing reproducibility or by portraying the life and work of prominent contemporary or historic figures such as Antoine and Marie de Lavoisier when introducing the concept of a scientific revolution. Alternatively, students may be asked to write short stories about serendipitous events or developments in science, such as Fleming’s discovery of penicillin.

### (iii) Thought-provoking questions and interdisciplinary dialogue.

Students are hungry for thinking intensely, critically, and broadly about how science works, what can be done to enhance reproducibility, and where ethical limits of scientific practice should be (102). This can be facilitated through group discussions that cover controversial issues in science or pertinent topics of public debate (65, 82, 87, 103). For instance, by asking open-ended questions in connection with peer critique, student teams could, e.g.,

∘ select among alternative experimental setups to collect evidence for the effect of a drug and judge the quality of the expected evidence,
∘ develop ideas for demonstrating redundancy in experimental design and justify their chosen strategies before their evaluating peers, and
∘ formulate and explain their train of thought about seemingly simple yet complex questions such as the following. What is science? Is there always a clear demarcation line between science and pseudoscience? What can we validly conclude from data sets? How do we cope with contradictions between what science makes possible and its moral limits?

Depending on respondents’ specialties, answers may be skewed toward the quantitative perspective for life scientists or a stronger inclusion of mixed-method aspects in the social or environmental sciences. Such perceived dichotomies are means to elicit engaged debates where student teams are asked to advocate for opposing viewpoints (82). Sparking interest and dialogue among participants from various science backgrounds and levels of experience, such conversations can stimulate cross-disciplinary fertilization (18, 104, 105) and thwart tendencies to think in narrow terms (67, 68, 72, 88, 91).

### (iv) Application and exploration through meaningful project work.

To help students synthesize and integrate their experiences from reflections and discussions and provide incentives for creative, self-directed, and in-depth exploration, graduate science programs should include frequent project work. In agreement with the instructor, students may be allowed to choose topics of their preference (e.g., “tell us about a paradigm shift in science that fascinates you and incorporate the concepts we covered over the duration of the course”). Providing incentives to apply the concepts learned to students’ own interests respects their diversity of talents (69), adds relevance (67, 70, 106), and motivates them to strive for their full potential (70, 84). This holds particularly true in experiential environments (66, 101) that allow students to discover their passions for their professional future. Besides academic research in laboratory rotations, this may be facilitated, e.g., during practicum experiences in the areas of communication, advocacy, community work, teaching, entrepreneurship, or science policy (19, 23, 29, 31, 36) (Tables 1 and 2).

### (v) Continuous communities of practice.

Critical thinking and scientific survival skills such as analysis, reasoning, evaluation, communication, and teamwork function like muscles: They need to be constantly exercised and nourished to stay in shape. Science education programs should therefore promote continuous, deliberate practice activities (107, 108) for students, as well as experienced practitioners, to foster sustained skill development. Periodically offered journal clubs, in combination with problem-solving exercises in a case study format (82), could be organized as competitive detective games, including prizes (82, 109). Focusing on error analysis and communication, interdisciplinary teams could be tasked with finding flaws in experimental design, logic, and statistical methods of a selected set of publications and engage in a scientific discourse for the best argumentation. Aiming to enhance participants’ motivation and willingness to think beyond the rim of their own field research, such collaborative formats might help to build confidence and generate a sense of collegiality and mutual appreciation for other fields of science (68, 94, 98, 110).

## TOWARD LONG-TERM OUTCOMES

Given concerns that graduate biomedical education is already taking too long (2, 10, 111), a potential criticism of the approach suggested here is that additional didactic instruction would increase the overall course load, reduce the time for research, and lengthen the time to a degree. In fact, the increasing time to independence for scientists was identified as a critical problem in the current biomedical research enterprise (2, 3, 30, 111). To avoid lengthening the time to a degree, we suggest that a significant portion of the currently mandatory, specialized courses that populate graduate curricula in the biomedical sciences be replaced with critical-thinking courses (Tables 1 and 2). Specialized knowledge necessary to productively pursue thesis work can be obtained through thesis-oriented electives or by self-study during the research-centered years of Ph.D. training (Table 2). Certainly, students who are highly interested in the specialized areas of their chosen research can be expected to largely self-master those fields with limited specific classroom instruction. Furthermore, we hope and posit that enhanced mastery of critical thinking tools and good research practices will lead students to make better choices for thesis research projects and carry out research more efficiently (112), which in turn could reduce training time (10, 11). In fact, our experience is that much of the time spent on Ph.D. research is used as students try to find projects and make them work. Better training in critical thinking could allow students to make better choices earlier, which could reduce the time to graduation (11). Hence, our proposal to reform graduate science education should, in our view, not lengthen training time and could, in fact, shorten it through enhanced efficiencies.

In the future, carefully validated, comprehensive observations and performance evaluations might provide further insights into potential strength and weaknesses of our approach; for instance, will the R^3^ program’s strong emphasis on critically creative reflection be able to attract an increasing number of learners from currently underrepresented groups into the biomedical disciplines (113, 114)? How will students with differing learning preferences perform? Will R^3^ graduates score above average in the quality of their scholarly products?

Broader impacts on the scientific enterprise level might only become visible after years of historical outcome data collection. Potentially, a more widespread adoption of the R^3^ approach to graduate science education across many disciplines might eventually contribute to an amelioration of some of the most prominent problems in the sciences, such as poor reproducibility, shoddy literature, and the rise in retracted publications (115–121). Although the causes of these problems are doubtlessly complex and causality is very difficult to establish, it is reasonable to assume that a better and more broadly trained scientific workforce could produce better science. This, in turn, could also help humanity confront some of the major challenges it is facing, including climate change, the failing green revolution, the threat of pandemics, and the need for new energy sources (122).

## THE R^3^ APPROACH TO “TEACHING AND THINKING SCIENCE”—A BRIDGE BUILDER AND CATALYST?

Watching students’ engaged discussions, which seemed unimpeded by disciplinary boundaries, we often observed the cross-fertilization, curiosity, and passion for science that educators long to see in their learners. These are the moments when being a science teacher is particularly rewarding. Some of the most impressive dialogues we witnessed between students took place in a class session on practical ethics: We talked about the moral dilemmas that can arise, e.g., in the field of genetic engineering, if scientists just focus on pushing the epistemic boundaries of what is “possible” but do not take adequate ethical considerations into account. It was gratifying to observe how eager the students were to think broadly and open their minds to horizons that went way beyond learning the next detail of, e.g., a pathogen’s life cycle, a biochemical pathway, or other minutiae that tend to dominate graduate biomedical science education. One of the students concluded a reflective essay with a quote from Henry Augustus Rowland, the first president of the American Physical Society: “Science may not be able to provide a complete code of ethics, but it does teach that every action carries a consequence—to be felt either by ourselves or by others, in our own time or the generations to come” (123). That is, our students remind us of the three R’s of good scientific practice: scientists’ responsibility to keep the potential outcomes and consequences of their work in mind and to make every effort possible that their experimental practice follows requirements for scientific rigor in order to enable reproducibility of their work (12, 124).

Reflecting on the many rich, engaged, and deep interactions we have had in our R^3^ courses thus far, it comes to mind how often we circled back to the ideas behind Albert Einstein’s famous quote at the beginning of this article. It gets right to the heart of what graduate science education should foster, i.e., scientists’ never-ending curiosity, the desire to think outside the box and to search for new insights. However, Einstein also reminds us that the privilege to engage in research comes with a strong ethical commitment to practice science rigorously (125). All practitioners across the disciplines must take Einstein’s admonition “not [to] conceal any part of what one has recognized to be the truth” as a constant appeal to do the best we can to ensure that our research and that of our students follows the norms of the three R’s.

Our responsibilities as educating practitioners do not end there. We need to think in bigger dimensions if we are to make a real difference in the current system. Initiatives such as the R^3^ track may help provide other educators with ideas on how to enhance critical-thinking strategies and to nurture passionate engagement in students at all stages of science education, not only the graduate level. Aspiring, broadly educated young scientists might act as open-minded catalysts and take the R^3^ way of thinking science with them after graduation. Committed science educators could collaborate across institutional boundaries and engage in high-quality educational research to agree on valid performance indicators and measure long-term learning outcomes to test the effectiveness of novel approaches to science education such as the R^3^ program. Equally important is, in our view, that the communities of science practitioners, educators, and philosophers reengage in regular dialogues and collaborate more actively to discuss joint efforts to fundamentally reform our science education system. These may include interdisciplinary, long-term communities of practice to exchange experiences in teaching science with philosophy elements in a practice-orientated, interest-sparking, and curiosity-sustaining manner. Finally, bridge-building colloquia should be held regularly to overcome the deepening communication divide that has grown between science and philosophy to revitalize the notion that science without philosophy is incomplete.

I have no special talents. I am only passionately curious.

—Albert Einstein
